# Simultaneous Determination of Ofloxacin and Ornidazole in Solid Dosage Form by RP-HPLC and HPTLC Techniques

**DOI:** 10.4103/0250-474X.73937

**Published:** 2010

**Authors:** Manisha Puranik, D. V. Bhawsar, Prachi Rathi, P. G. Yeole

**Affiliations:** Institute of Pharmaceutical Education and Research, P.G. Department of Quality Assurance, Borgaon (Meghe), Wardha-442 001, India

**Keywords:** RP-HPLC, HPTLC, ofloxacin, ornidazole, marketed formulation

## Abstract

The objective of this work was to develop and validate simple, rapid and accurate chromatographic methods for simultaneous determination of ofloxacin and ornidazole in solid dosage form. The first method was based on reversed phase high performance liquid chromatography, on Intersil C_18_ column (250 mm, 4.6 i.d.), using acetonitrile:methanol: 0.025M phosphate buffer, pH 3.0 (30:10:60 % v/v/v) as the mobile phase, at a flow rate of 1 ml/min at ambient temperature. Quantification was achieved with UV detection at 318 nm over a concentration range of 2-40 µg/ml for ofloxacin and 5-100 µg/ml for ornidazole. The mean retention time of ofloxacin and ornidazole was found to be 4.04 min and 5.83 min, 6.77 min (isomers), respectively. The amount of ofloxacin and ornidazole estimated as percentage of label claimed was found to be 100.23 and 99.61%, with mean percent recoveries 100.20 and 100.93%, respectively. The second method was based on TLC separation of these drugs using silica gel 60F_254_ aluminium sheets and dichloromethane:methanol:25% ammonia solution (9.5:1:3 drops v/v) as mobile phase. Detection was carried out at 318 nm over the concentration range of 20-100 ng/spot for ofloxacin and 50-250 ng/spot for ornidazole. The mean R_f_ value of ofloxacin and ornidazole was found to be 0.16 and 0.56, 0.78 (isomers), respectively. The amount of ofloxacin and ornidazole estimated as percentage of label claimed was found to be 100.23 and 99.61% with mean percent recoveries 100.47 and 99.32%, respectively. Both these methods were found to be simple, precise, accurate, selective and rapid and could be successfully applied for the determination of pure laboratory prepared mixtures and tablets.

Ofloxacin (OFL), (±)-9-fluro-3-methyl-10-(4-methyl-1-piperazinyl)-7-oxo-2,3-dihydro-7H-pyrido[1,2,3-de]-1,4-benzoxazine-6-carboxylic acid, exhibits good *in vivo* and *in vitro* activity against gram positive and gram negative bacteria[1]. Ornidazole (ORN), 1-chloro-3-(2-methyl-5-nitroimidazole-1-yl)propan-2-ol, is a derivative of nitroimidazole, with antiprotozoal and antibacterial properties. It is used in the prevention and treatment of infections due to anaerobic germs. The drug is specifically useful in abdominal and gynecological surgery[2].

Literature survey revealed that OFL is official in USP[3]. Several methods are reported for the determination of OFL by spectrofluorimetry[4] and HPLC by UV detection[5] and by fluorimetric detection[6]. ORN is not an official drug. Several methods are reported for determination of ORN by spectrophotometry[7], calorimetry[8], GC[9] in solid dosage form and in biological fluids. Few methods are also reported for simultaneous estimation of OFL and ORN by HPLC[10] and HPTLC[11].

In present paper, we report the HPLC and HPTLC methods for simultaneous determination of OFL and ORN in pure laboratory mixture and in tablet dosage form and their comparison. Combinations of ORN and OFL in solid dosage form are marketed in India as antiprotozoal and antibacterial. The marketed formulation selected for present study was Oflox OZ of Cipla Ltd., Jaipur, India.

ORN and OFL standards were supplied by Jenburkt Pharmaceutical Ltd., Sihor, Gujarat, India. HPLC grade water, methanol and acetonitrile were purchased from Loba Chemicals, Mumbai, India. Dichloromethane, ammonia and potassium dihydrogen phosphate used were of analytical grade.

HPLC system consists of JASCO (Japan) PU-1580 Intelligent pump, variable wavelength UV/Vis detector (Jasco UV 1575) and precision loop injector (Rheodyne, 20 µl). HPTLC system consists of Camag HPTLC (Germany) with Linomat V sample applicator, TLC scanner with CATS-4 software, Camag twin trough glass chamber, UV cabinet for visualization. A C_18_ Intersil column (250 mm, 4.6 i.d.) was used at ambient temperature. The mobile phase consisted of acetonitrile:methanol:0.025M phosphate buffer, pH 3.0 (30:10:60 %v/v/v) was prepared and pumped at a flow rate of 1 ml/min. The mobile phase was filtered through Whatman filter paper No. 41 and degassed prior to use. The elution was monitored at 318 nm. The injection volume was 20 µl.

Solutions of the test substance were applied to silica gel 60 F_254_ TLC plates. The plate was placed in a chromatographic tank previously saturated for 10 min with developing mobile phase; dichloromethane:methanol:25% ammonia solution (9.5:1:3 drops v/v). The plate was developed by normal vertical developing tank at ambient temperature for a distance of 90 mm. the spots were detected under a UV lamp and scanned densitometrically at 318 nm.

Accurately weighed amount of standards of OFL (100 mg) and ORN (250 mg) were transferred to 100 ml volumetric flask, separately and dissolved into and diluted up to the mark with methanol. The resulting solutions were sonicated for 15 min and filtered through Whatman filter paper No. 41. All solutions were prepared freshly.

An aliquot portion of the standard stock solution of OFL and ORN were further diluted with mobile phase to get the series of concentration of 2-40 µg/ml for OFL and 5-100 µg/ml for ORN. 20 µl of each solution was injected under operating chromatographic conditions described above. Calibration curve was constructed by plotting peak areas versus concentration and the regression equation was calculated.

The limit of detection was calculated and it was found to be 20 µg/ml and 50 µg/ml for OFL and ORN, respectively. Limit of quantification for OFL and ORN was found to be 60 µg/ml and 150 µg/ml, respectively.

An aliquot portion of standard stock solution of OFL and ORN were further diluted to get 0.01 µg/ml for OFL and 0.025 µg/ml for ORN. Each solution was applied as band ranging from 2-20 µl on TLC plate with Linomat V. The plate was developed in twin trough glass chamber under operating conditions described above. After development the plate was air dried and evaluated densitometrically at wavelength of 318 nm. Calibration curve was constructed by plotting peak areas versus concentration and the regression equation was calculated.

The laboratory mixture was prepared in the ratio of 1:2.5 w/w for OFL and ORN, respectively. From the stock solution aliquot portion was further diluted with mobile phase to get concentration 10 µg/ml for OFL and 25 µg/ml for ORN, for RP- HPLC method and 0.050 µg/ml for OFL and 0.125 µg/ml for ORN, for HPTLC method.

Twenty tablets were accurately weighed and finely powdered. An accurately weighed amount equivalent to 100 mg of OFL (equivalent to 250 mg of ORN) was transferred into 100 ml volumetric flask, along with 50 ml of methanol. The flask was mechanically shaken for 10 min; finally volume was made to mark with methanol, and filtered.

The stock solution was diluted with mobile phase to get concentration of 10 µg/ml for OFL (25 µg/ml for ORN) for RP- HPLC method. Further, the stock solution was diluted with methanol to get concentration of 50 µg/ml for OFL (125 µg/ml for ORN). Two microlitres of this solution was applied on TLC plate with Linomat V. The plate was developed in twin trough glass chamber under operating conditions described above and calculations were performed using peak area.

A solvent system that would give dense and compact spots with appropriate and significantly different Rf values was desired for quantification of combination by HPTLC. The mobile phase consisting of dichloromethane:methanol:25% ammonia solution (9.5:1:3drops v/v) gave the mean Rf value of 0.16 and 0.56, 0.78 (isomers), respectively for OFL and ORN. In HPLC conditions were optimized to obtain an adequate separation of eluted compounds. Initially, various mobile phase compositions were tried, but only acetonitrile:methanol:0.025 M phosphate buffer, pH 3.0 (30:10:60 % v/v/v) as the mobile phase at a flow rate of 1 ml/min at ambient temperature was found to give desired separation between OFL and ORN. The average retention times for OFL and ORN was found to be 4.04 min and 5.83 min, 6.77 min (isomers), respectively. Quantification was achieved in HPTLC as well as in HPLC with UV detection at 318 nm. Typical chromatograms for OFL and ORN for marketed formulations are shown in Figs.  1 and 2.

**Fig. 1:**
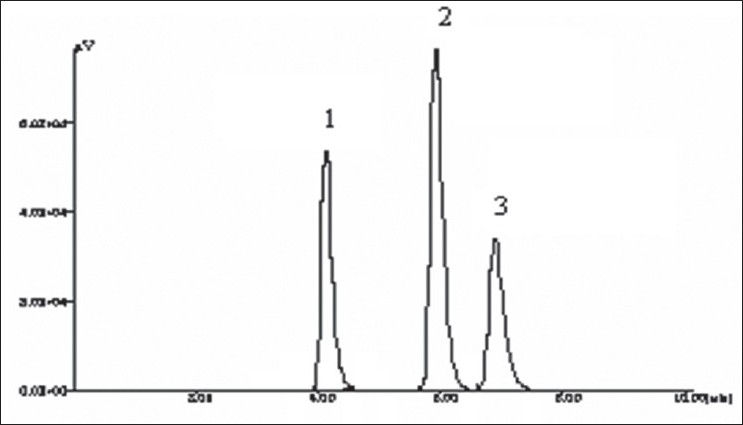
HPLC pattern of OFL and ORN Typical chromatogram of sample obtained from tablet containing OFL and ORN. HPLC retention times of OFL was found to be 4.04 min (1), for ORN I 5.82 min (2) and for ORN II 6.77 min (3).

**Fig. 2:**
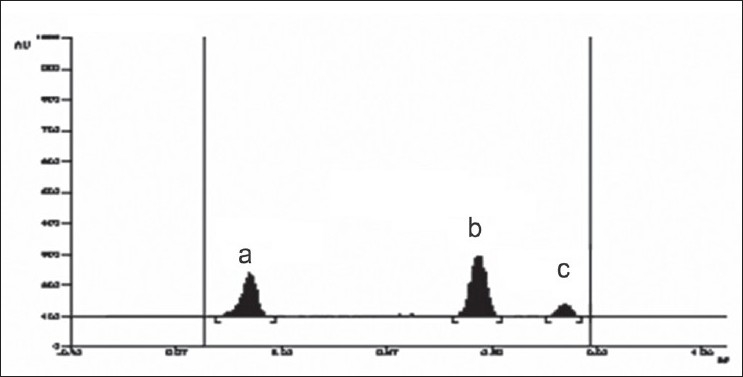
HPTLC pattern of OFL and ORN Typical chromatogram of sample obtained from tablet containing OFL and ORN. HPTLC R_f_ of OFL was found to be 0.16 (a), for ORN I 0.56 (b) and for ORN II 0.78 (c).

The developed method was validated in terms of linearity and range, limit of detection, limit of quantification, recovery study, and inter day study, intra day study and study by different analysts. The linear regression data in HPTLC showed a good linear relationship over a concentration range 50-250 ng/spot for OFL and 20-100 ng/spot for ORN. The correlation coefficients obtained were 0.9994 and 0.9989 for OFL and ORN, respectively. Repeatability of method was determined by six times spotting 10 µl of standard drug solution on TLC plate, after development the separated spots were scanned six times without changing position, measurement of peak areas was performed and from the peak areas the % RSD was determined. For OFL and ORN % RSD was found to be 0.0086 and 0.0063, respectively

System suitability tests are used to verify the reproducibility of the chromatographic system. To ascertain effectiveness of HPLC method, system suitability tests were carried out on freshly prepared standard stock solutions. The parameters obtained are shown in Table 1. The calibration curve was linear in concentration range of 2-40 µg/ml and 5-100 µg/ml, with regression 0.9994 and 0.9913, slope 0.6165 and 0.4314 for OFL and ORN, respectively.

**TABLE 1:** SYSTEM SUITABILITY PARAMETERS FOR RPHPLC

Parameters*	OFL	ORN I	ORN II
Retention time	4.04	5.83	6.78
Asymmetry	0.853	0.679	0.912
Capacity factor	2.46	3.97	4.83
Plate number	10,221	15,012	13,903
Resolution	-	10.06	4757
Selectivity	1.53	1.27	

* Mean of five determinations. OFL for ofloxacin; ORN I and II for isomer of ornidazole

Recovery studies were carried out to study accuracy and precision of the method. In HPTLC, these studies were carried out on plate at three levels i.e. multiple level recovery studies. Two microlitres of pre-analyzed sample preparation having concentration of 0.050 µg/µl for OFL and 0.125 µg/µl of ORN was applied three times on TLC plate as a band of 6 mm. These bands were spiked on plate with OFL and ORN standard stock solution and analyzed by the method. In HPLC, recovery study was carried out at 80%, 100% and 120% level. To the powder formulations the pure standard drugs were added and dilutions were made and analyzed by the method. The % recovery was calculated by using formula, % recovery = (T-A)/S×100 where, T is total amount of the drug estimated, A is the amount of drug contributed by tablet powder and S is the amount of pure drug added. The result of recovery studies for both OFL and ORN by both HPLC and HPTLC was found to be around 99-100%, indicating that the methods are free from interference from excipients (Table 2).

**TABLE 2:** ANALYSIS DATA AND RECOVERY STUDIES

Sample Code	Statistical Data*	% Label claim			
		HPLC Method		HPTLC Method	
		OFL	ORN	OFL	ORN
Standard Laboratory Mixture (% Estimation)	Mean	99.92	99.72	100.04	100.22
	SD	0.1361	0.4002	0.1581	0.4456
	RSD	0.0013	0.0040	0.0015	0.0044
Oflox OZ Tablet (% Label claim)	Mean	100.23	99.61	99.85	100.65
	SD	0.2468	0.3579	0.8602	0.6435
	RSD	0.0024	0.0035	0.0086	0.0063
Recovery Study (% Recovery)	Mean	100.20	100.93	100.47	99.32
	SD	0.4301	0.8105	0.5308	0.8900
	RSD	0.0043	0.0080	0.0052	0.0089

* Mean of six determinations; SD is standard deviation and RSD is relative standard deviation. OFL, ofloxacin; ORN, isomer of ornidazole.

Sample to sample precision and accuracy by both the techniques were evaluated using, three samples of three different concentrations, which were prepared and analyzed on same day. Day to day variability was assessed using three samples of three different concentrations analyzed on three different days, over a period of one week. The results show the accuracy and reproducibility of the assay. Thus, it was concluded that there was no significant difference on the assay, which was tested on an intra-day and inter-day basis. The % RSD values shows that proposed method provides acceptable intra-day and inter-day variation for OFL and ORN (Table 3).

**TABLE 3:** RESULT OF RUGGEDNESS STUDIES

Parameter	Statistical data	HPLC Method		HPTLC Method	
		OFL	ORN	OFL	ORN
Interday	Mean	100.28	100.11	100.28	99.97
	±SD	0.3160	0.6100	0.8673	0.8412
	RSD	0.0031	0.0061	0.0086	0.0083
	CV	0.3151	0.6093	0.8648	0.8370
Intraday	Mean	100.12	99.98	101.48	99.82
	±SD	0.4667	0.6501	0.6081	.8839
	RSD	0.0046	0.0065	0.0059	0.0088
	CV	0.4661	0.6502	0.5992	0.8854
Different analyst	Mean	100.20	100.15	99.87	99.86
	±SD	0.0400	0.7701	0.1768	0.9374
	RSD	0.0003	0.0076	0.0017	0.0093
	CV	0.0399	0.7689	0.1770	0.9347

^*^Mean of three estimations, SD is standard deviation and RSD is relative standard deviation; OFL, ofloxacin; ORN, ornidazole

From the above results it can be concluded that the HPTLC method is simple, accurate, most economic and less time consuming technique while RP- HPLC method is most accurate, precise, specific and very sensitive and can be used for routine analysis of OFL and ORN in their combined dosage form.
